# Using a Network Physiology Approach to Prescribe Exercise for Exercise Oncology

**DOI:** 10.3389/fnetp.2022.877676

**Published:** 2022-05-12

**Authors:** Gwendolyn A. Thomas

**Affiliations:** Department of Kinesiology, The Pennsylvania State University, University Park, PA, United States

**Keywords:** non-linear, person centered, exercise prescription, network physiology, exercise oncology

## Abstract

Current American College of Sports Medicine (ACSM) exercise guidelines for exercise oncology survivors are generic one-size fits all recommendations, which assume ideal or prototypic health and fitness state in order to prescribe. Individualization is based on the objective evaluation of the patient’s baseline physiological status based on a linear dose response relationship of endpoints. This is only a partial snapshot of both the acute and chronic responses exercise can provide. Each acute exercise session represents a unique challenge to whole-body homeostasis and complex acute and adaptive responses occur at the cellular and systemic levels. Additionally, external factors must be considered when prescribing exercise. Network physiology views the human organism in terms of physiological and organ systems, each with structural organization and functional complexity. This organizational approach leads to complex, transient, fluctuating and nonlinear output dynamics which should be utilized in exercise prescription across health states. Targeting health outcomes requires a multi-system approach as change doesn’t happen in only one system at a time or in one direction Utilizing a multi-system or person-centered approach, allows for targeting and personalization and understands and targets non-linear dynamics of change. Therefore, the aims of this review are to propose a paradigm shift towards a Network Physiology approach for exercise prescription for cancer survivors. Cancer treatment affects multiple systems that interact to create symptoms and disruptions across these and therefore, prescribing exercise utilizing both external daily factors and internal physiological networks is of the highest order.

## Introduction

Cancer diagnosis and treatment has a number of well documented physiological, psychological, and physical changes and challenges. A number of studies have demonstrated that cancer treatments are associated with either short-term or chronic dysfunction which includes an accelerated aging phenotype which can include multi-system declines (Uryga and Bennett, 2016; Chang et al., 2019). Regular exercise has been advocated for and has been shown to mitigate many of these systemic effects (McLeod et al., 2019). Resistance exercise (RE) and aerobic exercise (AE) are most often the types of exercise advocated for promoting health benefits in this population (Campbell et al., 2019). The field of exercise-oncology has expanded exponentially since the first review of exercise randomized control trials was published in the mid-1990s (Neil-Sztramko et al., 2019). The American College of Sports Medicine (ACSM) has a long history of recommending both aerobic and resistance exercise for improving health outcomes in cancer survivor populations and subsequent ACSM Roundtables have all included RCT based exercise recommendations (Schmitz et al., 2010; Campbell et al., 2019). Current recommendations for cancer assumes that fitness related outcomes improve with a dose-related response rate, which is linear. This approach, is rooted in a reductionist philosophy which is utilized across biomedical models. In the most recent ACSM roundtable on Exercise and Cancer Prevention and Control, recommendations for exercise guidelines and prescription was a fairly basic and universal one., The current recommendations call for performing RE at least two times per week, using at least two sets of 8–15 repetitions with at least 60% of one repetition maximum and 150 min of aerobic exercise at a moderate intensity or 75 min of vigorous intensity exercise spread across the week. Utilizing this heterogenous prescription has undoubtably yielded results across populations, however both the ACSM and partner organizations have highlighted the need to better understand how to truly optimize clinically relevant patient reported outcomes. (McNeely et al., 2006; Schmitz et al., 2010; Speck et al., 2010; Craft et al., 2012; Fong et al., 2012; Meneses-Echávez et al., 2015). One area of optimization is the consideration of the undulating nature of a cancer patient’s daily fatigue/pain/readiness and which would necessitate daily assessment to individualize exercise prescription.

**FIGURE 1:**
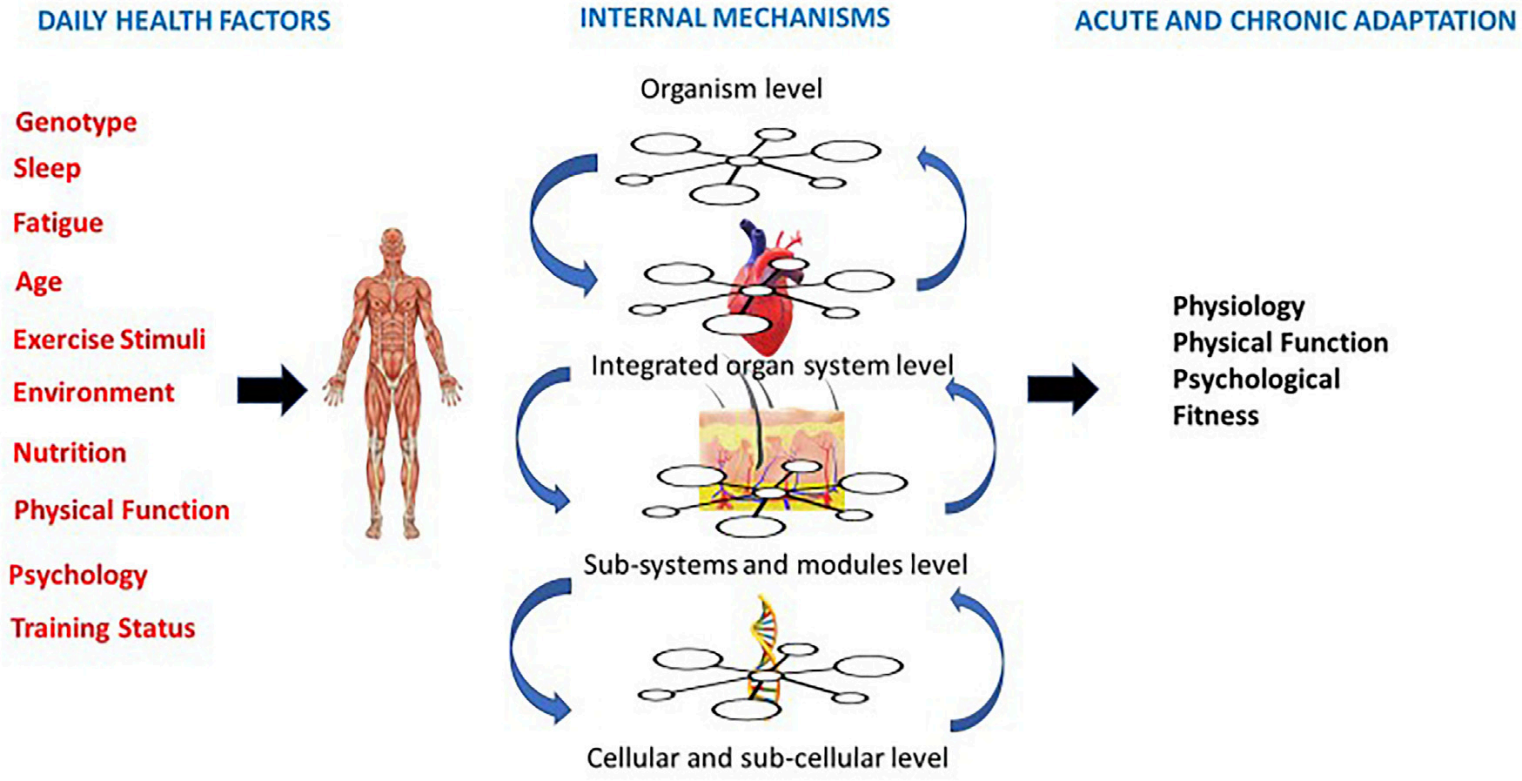
Person centered approach that centers on daily external factors, internal physiological mechanisms and both acute and chronic adaptations (adapted from Balague et al., 2020).

One reason for recommending this lack of precision it that results of randomized trials, focus on individual components of response and endpoints (i.e., fatigue, strength, lean muscle mass, fat mass). These endpoints only offer a partial snapshot of both the acute and chronic responses exercise can provide. Understanding how each acute (daily) exercise session represents a unique challenge to whole-body homeostasis is important to understand the complex adaptive responses that can occur at the cellular and systemic levels. Whole body exercise is a dynamic challenge that precipitates change in numerous cells, tissues and organs in response to each specific acute exercise challenge. It is important to understand that each acute session will offer an additive effect to facilitate eventual chronic change. Network physiology views the human organism in terms of physiological and organ systems, each with structural organization and functional complexity (Balagué et al., 2020). The outcomes of this approach are not one of dimensions and instead are unique and contextually dependent (Pol et al., 2020). Which leads to complex, transient, fluctuating and nonlinear output dynamics. Traditionally, clinical medicine frames itself around an approach defined by organ systems dynamics which is a reductionist approach to health and disease state (Pol et al., 2020). Current ACSM exercise guidelines are one-size fits all recommendations, which assume ideal or prototypic health and fitness state in which individualization is based on the objective evaluation of the patient’s baseline physiological status (Pross, 2012)This same approach has been applied to current exercise prescription guidelines used to treat and reduce symptoms across the cancer continuum. This prescriptive approach assumes that: (a) specific physiological adaptations occur with different types of exercise; (b) adaptations are dose responsive; (c) these adaptations will occur if exercise has adequate intensity (Balagué et al., 2020; Pol et al., 2020). However, exercise oncology studies using this prescription have often shown physiological, psychological and physical function non-responses, leading authors to conclude a given intervention is not effective and or not strong enough evidence to base future exercise recommendations on (Schmitz et al., 2010; Cheema et al., 2008). In contrast to using this approach to define fitness, new approaches focus on how diversity defines fitness (Pol et al., 2020). Specifically, by framing prescription around anetwork physiology perspective, fitness is able to adapt to daily socio-psycho-biological challenges (Balagué et al., 2020). This paradigm allows for a multi-system approach, as change doesn’t happen in only one system at a time or in one direction and requires a continuous process of diversification and specialization (Pross, 2012) (Figure 1). Utilizing a multi-system or network physiology approach, allows for targeting and personalization and understands how to optimally target non-linear dynamics of change (Balagué et al., 2020).

### Current Guidelines for Exercise Across the Cancer Continuum

Optimizing exercise training to improve post-treatment health and reduce risk of comorbid chronic disease/and recurrence is imperative for long term treatment and survivorship in the cancer continuum. However, much like those studies testing the benefits of exercise for healthy persons, the clinical guidelines for cancer patients closely adhere to the exercise guidelines of World Health Organization (2019) and American College of Sports Medicine (American College of Sports Medicine, 2018) (Piercy et al., 2018; Powell et al., 2019). Overall, the recommendations are similar for both healthy and clinical populations with little or no consideration of for physical function, physiological or psychological health or disease state.

Exercise oncology studies using prescriptions based on the national exercise guidelines have often shown physiological, psychological and physical function non-responses, leading authors to conclude a given intervention is not effective and/or not strong enough evidence to base future exercise recommendations on. (Campbell et al., 2019). This is not surprising, as using a reductionist approach to exercise prescription produces singular results. These results should be viewed with caution, as a non-response in one variable does not mean the individual is not benefiting in other outcomes (see Figure 1). Exercise outcomes in exercise oncology should thus be centered around the current health status of individuals and health-related goals should be considered as a spectrum, rather than one or two outcomes. Additionally, it has been hypothesized that an individual’s response to a given exercise stimulus may vary due to the direct effects of cancer treatments on physiological systems, the symptoms and side effects of cancer treatment, and demographic differences. (Schmitz et al., 2010; Sweegers et al., 2018). Most importantly, an individual’s ability to tolerate exercise may vary from day to day during and after active treatment. (Schmitz et al., 2010). Therefore, rather than broadly prescribing the same exercise protocol across patients, technology advances and our understanding of underlying mechanisms should be used to focus on clinical outcomes that are critical to improving health. Technology can also provide several important metrics to increase a professional’s ability to understand the context of responses and allow objective assessment of the exercise prescription within a daily readiness approach.

The principles of training derived from Exercise Physiology have remained largely impervious to the transdisciplinary and holistic insights emanating from complex systems approaches (Balagué et al., 2020; Pol et al., 2020). Often exercise physiologists rely on cardiovascular, respiratory, metabolic or neuromuscular endpoints that are based on the fragmentation of fitness in dimensions and sub-dimensions derived from reductionism (Balagué et al., 2020). This concept is most readily viewed in the view of V0^2^ max as a linear process that is trainable. Using this reasoning, every person who trained hard enough would be running the Boston marathon. This is of course not the case, as interactions change in time, both quantitatively and qualitatively. There is not a clear dose response relationship to VO (Chang et al., 2019) max among components. This is also evident in the limitations of current guidelines of exercise prescription in health and disease when reviewed on the basis of NPE. Research is also lacking to provide an understanding of exercising individuals as networked embedded systems and a clear absence of knowledge regarding the effects of exercise on the interactions among physiological systems.

Despite the adoption of a relatively homogeneous prescription approach, aerobic and strength training have been, for the most part, associated with benefits across a diverse range of populations (Bishop et al., 1999; Reid et al., 2010; Voisin et al., 2015; Flannery et al., 2019; Klil-Drori et al., 2020; Maestroni et al., 2020). On this evidence, it is assumed that a standardized, largely homogeneous exercise prescription that adopts a conventional approach is safe, efficacious, and therefore sufficient. Even though most studies present favorable results, systematic reviews and meta-analysis on exercise prescription point to the lack of high-quality studies showing the sustainability of standardized programs (Sørensen et al., 2006) and the need for personalizing the current recommendations (Zimmer et al., 2018). Current research is predominantly based on comparisons of group data means and evaluating quantitative changes of isolated variables in lab conditions. Such practices are clearly limiting the application of a precision exercise medicine approach (Balagué et al., 2020).

### A Network Physiology Understanding of Health and Disease

Current ACSM exercise guidelines are one-size fits all recommendations, which assume ideal or prototypic health and fitness state in which individualization is based on the objective evaluation of the patient’s baseline physiological status (Schmitz et al., 2010). Utilizing an approach that focuses on daily external factors to prescribe daily exercise, would allow for diversity to define fitness (Pol et al., 2020). Non-linear periodization offers an ideal framework in cancer populations due to the heterogeneity across cancers (i.e., medical history, various demographics, treatment type and duration, symptoms, comorbid chronic disease and demographics). This heterogeneity can have profound effects on daily motivation, readiness and also capacity for physical activity. (Sasso et al., 2015). Cancer treatment is associated with a myriad of physiological and psychological side effects, many of which create fluctuations in anxiety, depressive symptoms, fatigue, health related quality of life and physical function, all of which can change daily during the cancer continuum. (Craft et al., 2012; Jones and Alfano, 2013; Bower, 2014; Cvetković and Nenadović, 2016; Miller et al., 2016). Additionally, treatment can have side effects such as loss of muscle mass and increase in fat mass. (Kumar et al., 1997; Fearon et al., 2011; Elliott et al., 2017; Baracos and Arribas, 2018). Wildly disparate symptoms and side effects all support the premise that individualization that is contextually sensitive and meaningful is better to target multiple timeframes and an individual’s subjective and objective daily responses and should be considered as a prescriptive element of exercise prescription and subsequent daily training.

Non-linear periodization follows a network physiology of exercise approach, as it is a malleable method of autoregulation that considers assessment of an individual’s daily readiness is flexible nonlinear periodization. (Fleck, 2011). Flexible nonlinear periodization was developed by Fleck and Kraemer and uses the nonlinear training model framework, but changes training based on the daily readiness of a trainee to perform a specific training zone (Fleck, 2011). Daily training decisions are based upon several pieces of information that can include sleep, fatigue, physical function, and mood. In general, when performance or perceptions of ability to perform are higher, individuals pick more challenging sessions and on days perceptions are lower, individuals pick less challenging sessions. Therefore, the goal of flexible linear periodization is to alter the distribution of training to better align with an individual’s daily preparedness. Because training zones are not necessarily performed in a certain order due to self-selection.

### Revising Current Exercise Recommendations: Utilizing a Patient Centered Approach

Exercise prescription should thus focus on a Person-Centered Approach based in complex adaptive systems. Recent definitions of health reflect a dynamic interplay between the external environment and internal physiology (Speck et al. 2010; Sturmberg, 2018). Health can change in response to somatic conditions, social connectedness and emotional feelings (Sturmberg, 2018). Within this paradigm, the question becomes is this person in a stable or unstable health state and how can this stability be regained or maintained? This approach is centered in the emergent nature of a person and how objective and subjective variables inherently underly a person’s health and/or illness status.

The focus on disease management fails to see the other factors impacting health and impacting exercise outcomes (socio-economic factors) and thus the aim of prescription becomes a decrease in variability and improvements in quality of life. A daily exercise session needs to consider a patient’s health status beyond disease state and consider daily mood, nutrition, previous night’s sleep, illness (cold, cough, etc.), stress levels and the in the moment environment. These factors all individually contribute to how each exercise session will affect the physiological systems as a whole. Thus, a person-centered perspective that considers daily external variables and internal physiology (Figure 1) would combine both objective and subjective management and allow for a non-linear approach to prescription. This approach adapts the suggestions others have identified, that would allow for adapted, varied and sufficiently challenging individual exercise prescription.

**FIGURE 2:**
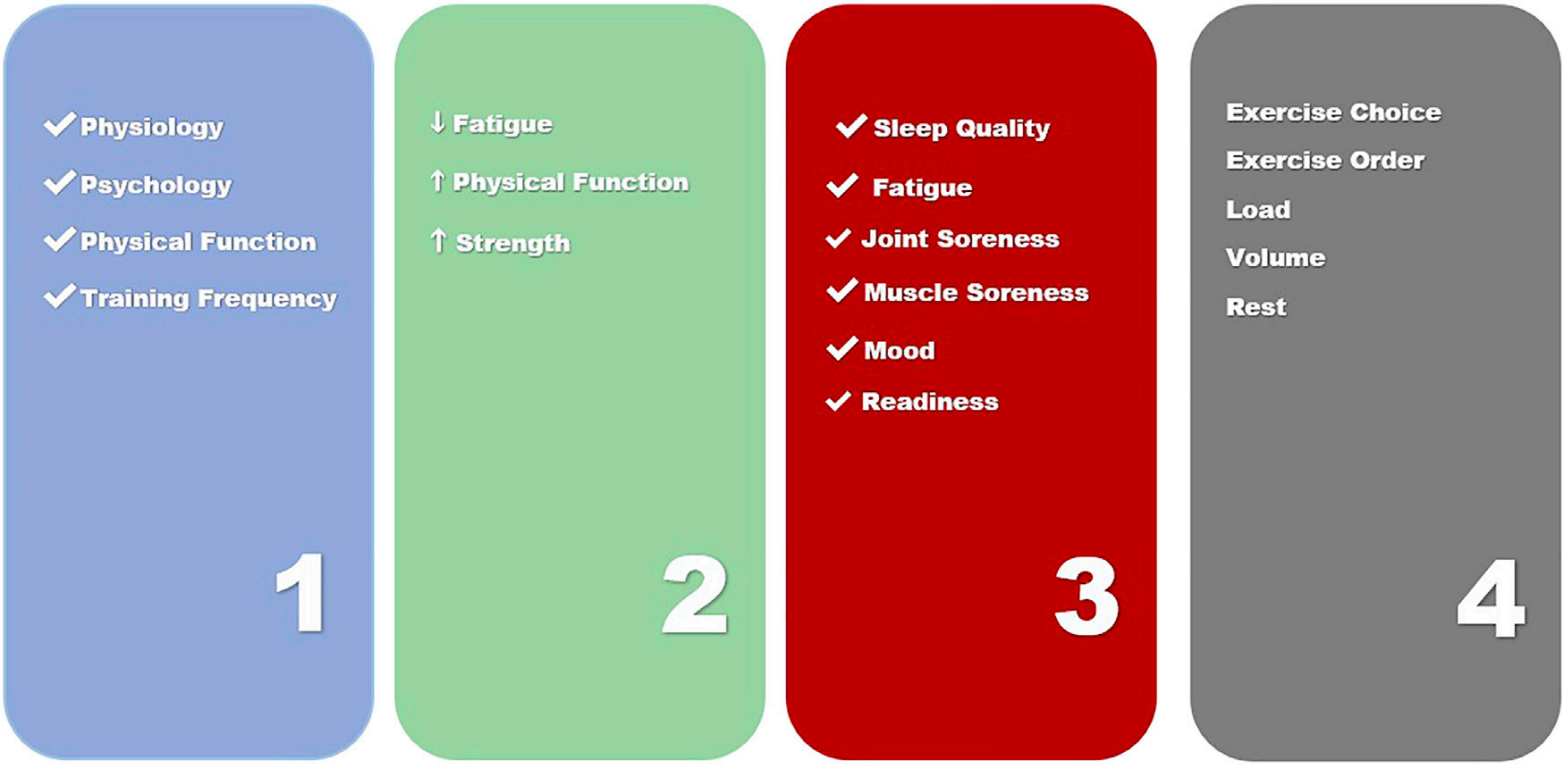
Person centered approach to daily exercise prescription.

Given the multitude of different responses that changing one of the four steps of exercise prescription (Figure 2) can elicit in both physiological and psychosocial adaptations, it appears the full therapeutic potential of exercise may be being masked by a lack of optimization and individualization in oncology settings. Optimization and individualization are widely employed in exercise science and the practice involves purposefully adjusting training to center around measurements of both training and non-training related stressors (sleep quality and quantity, nutrition, mood, illness, fatigue). The concept of autoregulation is one that describes an emergent process that can be used to systematically individualize exercise training (Haley et al., 2019). Autoregulation is based on an individual’s performance and perceptions of their ability to perform. Historically, autoregulation is applied in one of three ways, to adjust intra-training loads, as a weekly progression or more recently to select a daily set and repetition schemes prior to exercise based on current mood, sleep or fatigue levels. Current research has demonstrated that autoregulation of training may be superior to training strategies that employ predetermined loading strategies for such training goals as strength and increased lean body mass and is believed to be a framework that can enhance other physiological and psychological outcomes (Mann et al., 2010; Fleck, 2011; Colquhoun et al., 2017; Sturmberg, 2018; Greig et al., 2020). A method of autoregulation, non-linear periodization has recently been proposed for aerobic exercise in cancer populations (Sasso et al., 2015). Such an approach could also be useful in prescribing resistance exercise, as a nonlinear approach could prevent overtraining and plateaus in a population with multiple symptoms requiring highly context specific individualization (Schwartz, 2000; Mcnamara and Stearne, 2010; Klijn et al., 2013; Duijts et al., 2014). Nonlinear periodization is implemented by utilizing daily and weekly alterations in volume and intensity (Bower et al., 2000). Exercise programs are periodized by properly combining and monitoring the four key training principles of specificity, overload, variation and progression. Additionally, periodization of training is accomplished by implementing planned changes to any of the acute training variables to bring about continued and optimal fitness gains. Based on these factors, the prescription of exercise becomes a dynamic process. Optimized prescription must be based on the measurement of a patient’s performance or perceived ability to perform and be responsive to the changing levels of adaptation and functional capacities to be systematically individualized (Roy, 2009; Jim et al., 2011; Klemp et al., 2016).

### Person Centered Exercise Prescription Steps

Over 50 years of exercise research has informed practice in athletic and general populations, however these findings have rarely been used in clinical trials and populations (Campbell et al., 2019) Athletic performance has been informed by this practice which has been able to adjust and improve precision and personalization of dosing, scheduling and minimization of injury through rapid advances in exercise science (Jones and Alfano, 2013; Sasso et al., 2015). Given that the current non-specific exercise prescription in exercise oncology research and the resulting lack of individualization this current consensus guidelines it utilizes, it is important to understand how refined prescription and individualization can optimize outcomes in cancer populations trials. This could move current t exercise oncology research beyond assessments on based on quantitative benchmarks, which result in conclusions drawn from isolated variables and functions. These benchmarks provide little information about the coordinated activity of physiological systems and are not sufficiently responsive to daily training effects. This exercise prescription is based on the tenets of reductionism, which are centered around a three-step process. A needs analysis, the acute program variables (in Resistance Exercise) or the FITT principle (in Aerobic Exercise) and an application of the key training principles (specificity, overload, variation and progression. It is believed that application of these key training principles are what will produce the desired outcomes (ie strength, hypertrophy, muscular endurance, power, producing change in biomarkers, decreased depression, decreased anxiety, increased quality of life and physical function) (Wolin et al., 2012). However, given the multitude of different responses that changing one of the three steps of exercise prescription can elicit in both physiological and psychosocial adaptations, it appears the full therapeutic potential of exercise may be being masked by a lack of optimization and patient centered individualization in oncology settings.

A person-Centered Exercise Prescription offers an ideal framework in cancer populations due to the heterogeneity across cancers (i.e., medical history, various demographics, treatment type and duration, symptoms, comorbid chronic disease and demographics). This heterogeneity can have profound effects on daily motivation, readiness and also capacity for physical activity (Sasso et al., 2015). Cancer treatment is associated with a myriad of physiological and psychological side effects, many of which create fluctuations in anxiety, depressive symptoms, fatigue, health related quality of life and physical function, all of which can change daily during the cancer continuum (Craft et al., 2012; Jones and Alfano, 2013; Bower, 2014; Cvetković and Nenadović, 2016; Miller et al., 2016). Additionally, treatment can have side effects such as loss of muscle mass and increase in fat mass (Kumar et al., 1997; Fearon et al., 2011; Elliott et al., 2017; Baracos and Arribas, 2018). Wildly disparate symptoms and side effects all support the premise that individualization is better to target multiple timeframes and an individual’s subjective and objective daily responses and should be considered as a prescriptive element of exercise training.

Therefore, exercise prescription in the cancer continuum should be centered around a four-step process. This four-step process is a combination of understanding how acute exercise sessions combine to affect chronic change (Figure 2).

1) Completion of a Person-Centered Needs Analysis
2) Person Centered Exercise Goals Co-Created with Patient
3) Daily Readiness Assessment and Score to Inform Acute Exercise Prescription
4) Daily Exercise Prescription Based on a Daily Readiness Score

### Repeat Steps 3 and 4 Daily to Prescribe Exercise to Inform Chronic Response

This Person-Centered approach to autoregulation is based in flexible nonlinear periodization and considers an individual’s daily readiness to prescribe acute exercise. This approach uses the nonlinear training model framework but changes daily training based on the daily readiness of a trainee to perform in a specific training zone (Fleck, 2011). Daily training decisions are based upon several pieces of information that can include sleep, fatigue, physical function, and mood. In general, when performance or perceptions of ability to perform are higher, individuals pick more challenging sessions and on days perceptions are lower, individuals pick less challenging sessions and activities. The ultimate goal of such a prescription is to alter the distribution of training to better align with an individual’s daily preparedness. and allow the person to be the co-creator of their daily training session. Because training zones are not necessarily performed in a certain order due to daily fluctuations, intensity or volume does not follow a consistently increasing or decreasing pattern. This distinction is very important because it will allow clinical populations to focus on four key elements:

-more efficient recovery patterns (ie stress to recovery ratio)
-add more or less volume per week (dependent on daily fatigue or other symptoms)
-increase training frequency (by responding to in the moment feeling)
-decrease plateaus that lead to discontinuation of exercise (increased adherence)

## Conclusion

Despite over 50 years of research into manipulation of non-linear exercise program design, these exercise prescriptions are not currently used in exercise oncology practice and research. To date, only one study had examined nonlinear periodized training in a clinical population (Mann et al., 2010). The study demonstrated superior results in this population, which warrants further examination. An individualized resistance exercise programs utilizes the four-step process of a needs analysis, the acute program variables and key exercise principles (Figure 2). Specific exercise prescription utilizing autoregulation should be considered across multiple timeframes to include within session, meta-session and across program levels. The most efficacious resistance and aerobic exercise approach for an individual cancer survivor or patient is still being elucidated and must include constant monitoring of an individual’s subjective and objective responses. This review has provided a framework to individualize exercise prescription in exercise oncology research. Due to advances in technology that enable feasible, accurate and reliable methods to measure physiological, performance and psychological data, novel methods such as flexible nonlinear periodization can be implemented more readily in exercise oncology and those prescriptions can be used to inform best practices for survivors and patients across the continuum.

Understanding symptoms and side effects of cancer treatment requires an understanding of complex, nonlinear systems within individuals with dynamic day to day and in the moment states. In order to support targeted outcomes across systems we must improve our prescriptive pproach to allow for an understanding of nonlinear health This understanding allows adaptations to daily contexts and creates training programs that have less stress and less risk of attrition (Balagué et al., 2020) A non-linear state (the cancer continuum), requires a form of exercise that is easily adaptable and readily programmable to dynamic change. (Figure 1). By emphasizing true individualization to effectively mitigate these symptoms, we may increase adherence and enhance outcomes across side effects. By furthering emphasis on such approaches we will allow a focus on integrative approachs that allows understanding of multi-system outcomes. Further investigations and clinical trials should focus on understanding how targeting multi-system responses can facilitate health benefits.
